# Breast cancer screening adherence after multigene panel testing among women with pathogenic variants in moderate-risk genes or with empirically increased breast cancer risk

**DOI:** 10.1007/s10549-026-08020-5

**Published:** 2026-07-10

**Authors:** Jacob G. Comeaux, Natalia Gutierrez, Charité N. Ricker, Qi Nie, Averi Nguyen, Ivan Garcia, Emmeline Y. Chang, Rebecca M. Waggoner, Susan Groshen, Caryn Lerman, Darcy Spicer, Julie O. Culver

**Affiliations:** 1 Keck School of Medicine, University of Southern California, Los Angeles, USA; 2 Los Angeles General Medical Center, Los Angeles, USA; 3 USC Norris Comprehensive Cancer Center, University of Southern California, Los Angeles, USA

**Keywords:** Breast cancer, Breast MRI, Mammogram, Screening adherence, Genetic testing, Germline pathogenic variant

## Abstract

**Purpose:**

To identify factors associated with adherence to breast cancer (BC) screening among women with a pathogenic or likely pathogenic (P/LP) variant in a moderate-risk gene, *ATM*,* BARD1*,* CHEK2*,* RAD51C*, and *RAD51D* or with empiric lifetime risk ≥ 20%.

**Methods:**

We reviewed medical records of women without breast cancer who underwent genetic counseling and testing and were advised to undergo annual breast MRI and mammography. Adherence to screening for up to three years was compared between women with a P/LP variant in a moderate-risk gene and women with an elevated empiric risk of 20–40% by the Tyrer-Cuzick model.

**Results:**

The study population included 44 women with P/LP variants (P/LP group) and 117 with lifetime BC estimates of 20–40% (Empiric group). Within one year, the P/LP group had significantly higher rates of screening than the Empiric group with 58% vs. 37% obtaining breast MRI and 88% vs. 55% obtaining mammograms. Time-to-event analysis demonstrated the P/LP group initiated screening significantly sooner than the Empiric group. After adjusting for having family history of BC or personal history of non-BC, the P/LP group was 3.18 and 7.04 times more likely to obtain breast MRI and mammograms, respectively. Adherence declined in both groups in the second and third year of follow-up.

**Conclusion:**

Women with P/LP variants were more adherent to BC screening than those with empiric risk, underscoring the role that genetic test results may play in screening behavior. Future efforts should focus on understanding the barriers to screening and improving long-term adherence.

## Introduction

Breast cancer (BC) risk assessment is an essential process for tailoring screening strategies that has advanced considerably with the availability of multigene panel testing (MGPT) and empiric BC risk models [1, 2]. Criteria for MGPT provided by the National Comprehensive Cancer Network (NCCN^®^) have also continued to expand, thereby identifying more women with pathogenic and likely pathogenic (P/LP) variants [1].

In addition to high-risk BC genes *BRCA1*,*BRCA2*, and *PALB2*, MGPT typically includes moderate risk BC genes such as *ATM*,* CHEK2*,* BARD1*,* RAD51C*, and *RAD51D*. Women with a P/LP variant in a moderate-risk gene have lifetime BC risks ranging from 20 to 40% [1, 3] compared to the population risk of 13% [4]. Women who test negative may still be at moderate risk for BC based on the Tyrer-Cuzick empiric risk model, which takes into consideration age, family history of BC, benign breast disease, reproductive factors, breast density, and body mass index [5].

The addition of breast MRI to mammography aids in early detection of BC in women with a strong family history of BC or a P/LP variant in *BRCA1*,* BRCA2*, or *PALB2* [1, 6–8]. In women at moderate risk for BC, a simulation model conducted by Lowry et al. showed that initiating annual MRI screening at age 30–35 in *ATM* and *CHEK2* P/LP carriers reduced BC mortality by more than 55% [9], leading to the NCCN Guideline recommendations for women with moderate-risk P/LP variants to consider annual breast MRI screening in addition to annual mammogram [1], typically beginning at age 30–40. Bilateral risk-reducing mastectomy (RRM) is typically not recommended for women in this risk category [1].

The Tyrer-Cuzick model is widely utilized for empiric BC risk assessment to identify patients with a lifetime BC risk exceeding 20%. For such women, NCCN guidelines recommend breast MRI screening, starting at age 40, or 10 years younger than the youngest BC diagnosis in the family [2, 7]. Radiology centers increasingly use the Tyrer-Cuzick model at the time of mammography to identify patients with ≥ 20% risk who may benefit from MRI screening, offering an avenue to dedicated breast screening care [10].

While women with a moderate-risk P/LP variant may have the same risk level as those with family history alone, knowledge of a genetic marker may provide extra motivation for screening. The limited studies of women with moderate-risk P/LP variants have included very few individuals [11–13] or have relied only on patient recall [11, 12, 14]. Therefore, the screening patterns in those with P/LP in moderate-risk genes is understudied, as is the effect of knowledge of the variant.

We hypothesized that women with a moderate-risk P/LP variant are more likely to adhere to mammogram and breast MRI screening recommendations in the first year following genetic counseling (GC) than those who test negative but have a 20–40% lifetime BC risk. We also sought to understand whether screening adherence declined over time and was impacted by demographic or clinical characteristics. Our goal is to help providers understand factors associated with BC screening adherence so that future efforts can be made to improve early detection among women at increased risk.

## Methods

### Study population and study design

Our study population included women who underwent genetic counseling and MGPT between January 2021 and March 2024 and had GC at the USC Norris Cancer Hospital, part of the Keck Medical Center of USC (USC Norris), a university medical center, or Los Angeles General Medical Center (LA General), a safety-net hospital. The study time frame began in 2021 to reduce the influence of the COVID-19 pandemic on cancer screening. We received approval from our Institutional Review Board for our chart review protocol.

Women in the study had a moderate risk of developing BC based on either (1) a P/LP variant in the genes *ATM*,* BARD1*,* CHEK2*,* RAD51C*, or *RAD51D* or (2) a lifetime BC risk estimate ranging from 20 to 40% as calculated by the Tyrer-Cuzick empiric risk model after negative MGPT. The 20–40% moderate risk range for the Empiric Group was defined by the minimal threshold at which breast MRI is recommended as well as the highest lifetime BC risk for the included genes per the NCCN Guidelines during the study period [3].

Women were eligible if they were 30–75 years old, had no personal history of BC or any metastatic cancer, and if their GC recommended mammography and screening breast MRI to begin at their current age. Almost all of the patients in the study had pre-test GC by a board-certified genetic counselor and provided informed consent for testing. All patients in this study met with genetic counselors for post-test GC during which risk assessment and recommendations for screening were provided. We identified women in the P/LP Group by querying our internal Progeny software database (Progeny version 10.6.2.0) for women testing positive for the moderate-risk BC genes for which NCCN Guidelines recommended breast MRIs, including *ATM* and *CHEK2* starting version 1.2015 (3/30/15), *BARD1* starting NCCN version 1.2021 (9/8/2020), and *RAD51C*,* RAD51D* starting version 1.2023 (9/7/2022). We confirmed by medical record review that the eight women with *CHEK2* moderate-risk variants I157T, S428F, or T476M were recommended to undergo enhanced screening. Patients were typically given age-adjusted lifetime BC risk estimates using the ASK2ME or CanRisk models [15, 16].

**Fig. 1:**
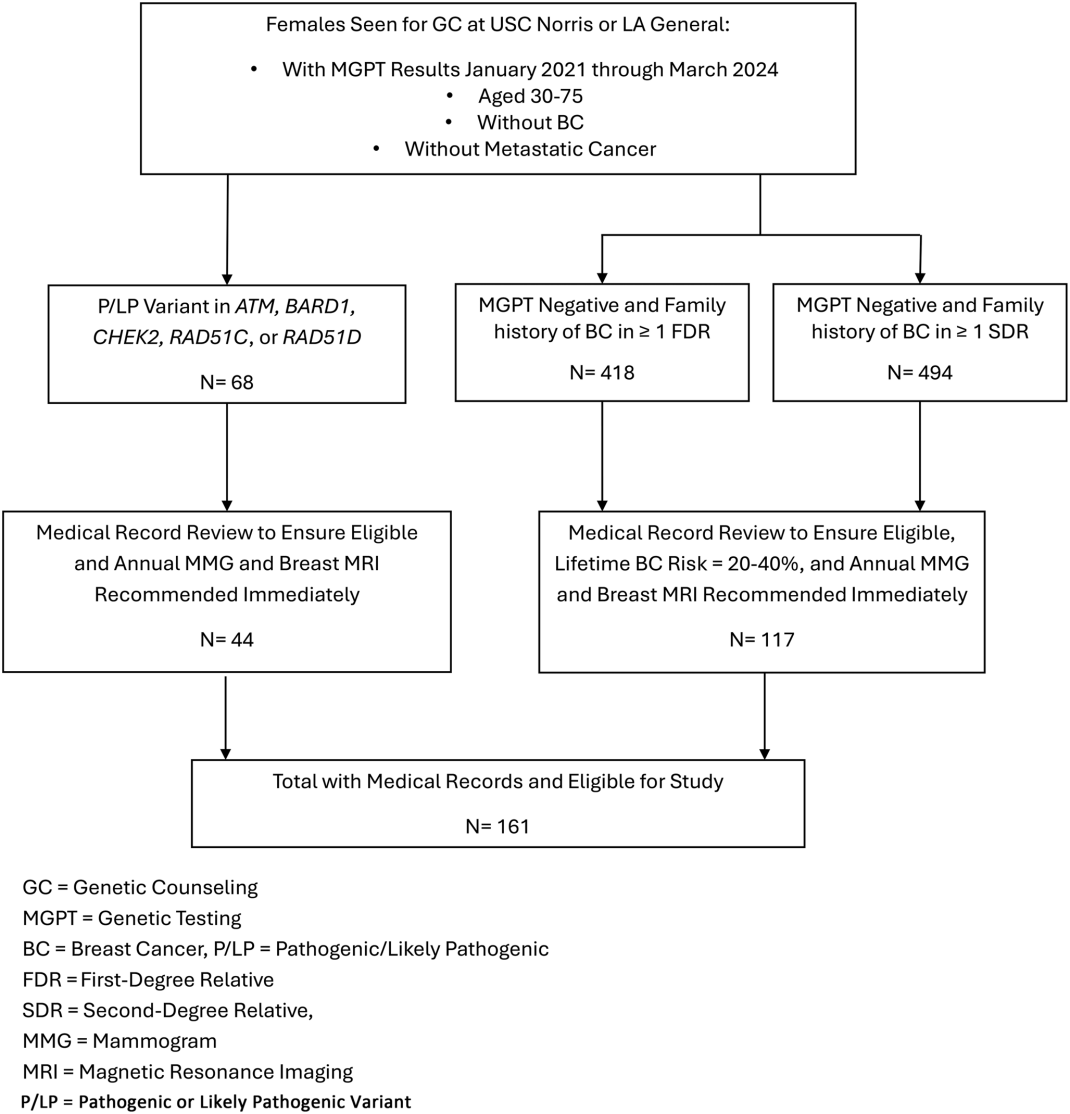
Study participant selection among patients undergoing cancer genetic counseling

We identified the Empiric group by searching the Progeny database for women who tested negative on MGPT who also had a family history of BC in first- or second-degree relatives. We then reviewed clinical GC documentation in the electronic medical record (EMR) to ensure that the Tyrer-Cuzick lifetime BC risk estimate [5] provided ranged from 20 to 40% (Fig. 1).

### Data collection

We reviewed the EMR, capturing demographics, family history, and dates of mammograms and breast MRIs after GC recommendations, including imaging performed at our facilities and any available outside medical records. Data was stored in a Redcap database (version 15.9.1). Biopsy results, breast cancer diagnoses, and risk-reducing mastectomy (RRM) data were also collected and analyzed. Since USC Norris patients are privately insured, their insurance type was categorized into Health Maintenance Organization (HMO) or Preferred Provider Organization (PPO). Due to insurance type, HMO patients are required to be seen by providers within their healthcare network and need an authorization to be seen at our facility whereas PPO patients may self-refer for care at our facility. LA General patients meet criteria for public and hospital programs such as Medicare, Medicaid, or Ability-to-pay and receive care at no-or-reduced costs.

We defined follow-up period as the time from GC recommendation to the date of the most recent encounter in the EMR. Women were eligible for analysis if they had a full year of follow-up in the EMR after GC recommendations and/or had evidence of at least one mammogram or breast MRI following GC recommendations. Women were eligible for year-two and year-three analysis if their follow-up periods were at least two and three years, respectively, or if they had evidence of at least one BC screening in the second or third year of analysis, respectively. To measure continuous adherence, women were defined as adherent to either screening in years two and three only if they were also adherent in each prior year. For example, adherence to MRI for three years would mean that a patient received an MRI in year one, year two, and year three. However, if a patient with three years of follow up in the EMR was not adherent to MRI screening in year one, then she was considered non-adherent in year two and three as well. Using this method allowed us to study consistency of screening adherence over time.

### Statistical analysis

Continuous variables were compared using the Mann-Whitney U test, and categorical variables were assessed with the Chi-squared or Fisher’s exact test when expected cell counts were less than 5. Variables significant in univariable analyses were further evaluated using logistic regression to quantify the direction and magnitude of associations between screening and study groups (P/LP vs. Empiric), with both unadjusted and adjusted models reported. To reduce bias, we included variables in the logistic regression model that significantly differed between study groups. Time to screening initiation was analyzed using cumulative incidence functions (CIFs), and group differences were assessed with Gray’s test. To evaluate whether adherence to breast MRI or mammography changed over time for the entire study population we used generalized estimating equation (GEE) logistic regression models. This approach enabled formal testing of temporal trends in adherence while appropriately accounting for the non-independence of repeated observations. All analyses were performed in R (version 4.5.0) [17], and the critical value was set at 0.05.

## Results

### Demographics by study group

Our study population of 161 women (Fig. 1) was diverse; 47% were non-Hispanic White, 34% Hispanic, 13% Asian, and 6% had other or mixed ancestry (Table 1). The median age of our group was 45 years old. There were 44 women in the P/LP group and 117 in the Empiric group. The P/LP group included women with a P/LP variant in *ATM* (17), *BARD1* (3), *CHEK2* (18), *RAD51C* (3), and *RAD51D* (3).

The P/LP and Empiric groups did not differ from each other by race/ethnicity, age, hospital site, or lifetime BC risk. However, more women in the Empiric group had a first-degree relative (FDR) with BC and more women in the P/LP group had a personal history of non-BC (Table 1).

**Table 1:** Demographics by study group

Category	Variable	Study group		Total *N* = 161	*p*
		Empiric *n* = 117 *n* (%)	P/LP *n* = 44 *n* (%)		
Race/Ethnicity	White Non- Hispanic	52 (44.4)	23 (52.3)	75 (46.6)	0.351
	Hispanic	38 (32.5)	17 (38.6)	55 (34.2)	
	Asian	17 (14.5)	4 (9.1)	21 (13.0)	
	Black	4 (3.4)		4 (2.5)	
	Mixed/Other	6 (5.1)		6 (3.7)	
Age	Median (IQR)	44.7 (38.9 to 52.5)	45.9 (41.5 to 53.1)	45.0 (39.6 to 52.5)	0.254
Hospital Site	LA General	32 (27.4)	13 (29.5)	45 (28.0)	0.937
	USC Norris	85 (72.6)	31 (70.5)	116 (72.0)	
Insurance Type^1^	HMO	10 (8.5)	2 (4.5)	12 (7.5)	0.748
	LA General	32 (27.4)	13 (29.5)	45 (28.0)	
	PPO	75 (64.1)	29 (65.9)	104 (64.6)	
Cancer History other than BC^2^	No	112 (95.7)	33 (75.0)	145 (90.1)	< 0.001
	Yes	5 (4.3)	11 (25.0)	16 (9.9)	
FDR with BC	No	26 (22.2)	33 (75.0)	59 (36.6)	< 0.001
	Yes	91 (77.8)	11 (25.0)	102 (63.4)	
Lifetime BC Estimate (%)	Median (IQR)	25.0 (21.6 to 30.4)	25.2 (21.6 to 31.6)	25.0 (21.6 to 30.4)	0.867

FDR: First-Degree Relative; BC: Breast Cancer; P/LP: Pathogenic or Likely Pathogenic Variant

^1^ Insurance Type: HMO = Health Maintenance Organization, PPO = Preferred Provider Organization, LA General = safety net hospital insurance plans combined

^2^ In the Empiric group, the cancer history included cervix (1), lymphoma (1), melanoma (2), and sarcoma (1). In the P/LP group, the cancer history included kidney (1), melanoma (1), ovary (2), pancreatic neuroendocrine (1), pituitary adenoma (1), sarcoma and rectum (1), thyroid (1), thyroid and melanoma (1), and uterus (2)

### Screening adherence in year 1

**Fig. 2:**
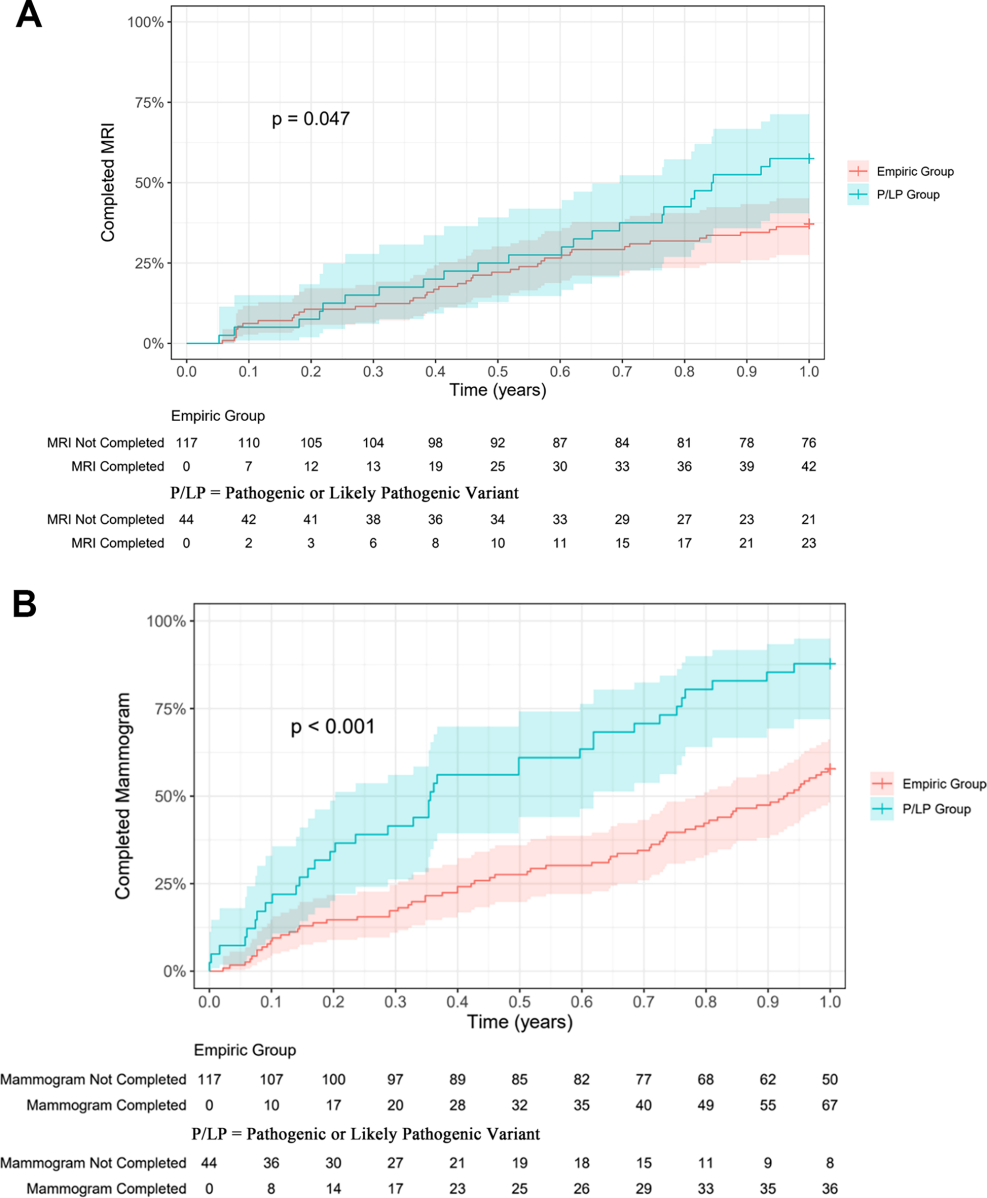
**A**. Time-to-event analysis showing breast MRIs obtained within year one in both study groups. **B**. Time-to-event analysis showing mammograms obtained within year one in both study groups

In the first year of follow up, both MRI and mammogram adherence was higher in the P/LP group than the Empiric group (Table 2). Significantly more women in the P/LP group (23/44, 58%) obtained MRI within one year compared to the Empiric group (42/117, 37%) (*p* = 0.04). Similarly, significantly more women in the P/LP group (36/44, 88%) obtained a mammogram within one year compared to the Empiric group (67/117, 58%) (*p* = 0.001). As shown in Fig. 2A, B and a time-to-event analysis showed that women in the P/LP group obtained mammograms more quickly (*p*<0.001) and had a higher cumulative probability of MRI screening (*p* = 0.047) than those in the Empiric group.

**Table 2:** Breast screening adherence by study group

Category	Total Available for Analysis	Variable	Study group		Total *N* = 161	*p*
			Empiric *n* = 117 *n* (%)	P/LP *n* = 44 *n* (%)		
**Year 1**						
MRI in year 1 Completed	153	No	71 (62.8)	17 (42.5)	88 (57.5)	0.040
		Yes	42 (37.2)	23 (57.5)	65 (42.5)	
Mammogram in year 1 Completed	157	No	49 (42.2)	5 (12.2)	54 (34.4)	0.001
		Yes	67 (57.8)	36 (87.8)	103 (65.6)	
**Year 2**						
MRI in years 1 and 2 Completed	93	No	47 (65.3)	13 (61.9)	60 (64.5)	0.980
		Yes	25 (34.7)	8 (38.1)	33 (35.5)	
Mammogram in years 1 and 2 Completed	95	No	35 (48.6)	5 (21.7)	40 (42.1)	0.042
		Yes	37 (51.4)	18 (78.3)	55 (57.9)	
**Year 3**						
MRI in years 1, 2, and 3 Completed	46	No	28 (80.0)	7 (63.6)	35 (76.1)	0.418
		Yes	7 (20.0)	4 (36.4)	11 (23.9)	
Mammogram in years 1, 2, and 3 Completed	51	No	23 (57.5)	3 (27.3)	26 (51.0)	0.098
		Yes	17 (42.5)	8 (72.7)	25 (49.0)	

P/LP: Pathogenic or Likely Pathogenic Variant

We also performed a logistic regression model which included FDR status and non–BC cancer history because they were associated with the study group and are clinically relevant covariates that could potentially confound screening behavior. As indicated in Table 3, women in the P/LP group were 2.29 times as likely to obtain MRI in one year than the Empiric group (*p* = 0.027), and when adjusting for personal history of non-BC and FDR with BC, women in the P/LP group were 3.18 times as likely to obtain MRI (*p* = 0.012). Women in the P/LP group were 5.27 times as likely to obtain mammogram in year one than the Empiric group (*p* = 0.001), and when adjusting for personal history of non-BC and FDR with BC, women in the P/LP group were 7.04 times as likely to obtain mammogram (*p* = 0.002). Thus, in this model, the P/LP variant has a stronger effect on screening adherence in the first year and FDR status was not associated with the likelihood of undergoing MRI (*p* = 0.78) or mammography (*p* = 0.70) within one year. Similarly, prior non-BC cancer history was not associated with adherence to breast MRI (*p* = 0.078) or mammography (*p* = 0.086).

**Table 3:** Univariable and multivariable analysis of the effect of moderate-risk variant on breast screening adherence

Variable	Comparison	Unadjusted OR, 95% CI	*p*-value	Adjusted^1^ OR, 95% CI	*p*-value
MRI in year 1 Completed	P/LP Group vs. Empiric Group	2.29 (1.10–4.82)	0.027	3.18 (1.32–8.13)	0.012
Mammogram in year 1 Completed	P/LP Group vs. Empiric Group	5.27 (2.08-116.18)	0.001	7.04 (2.22–28.80)	0.002

P/LP: Pathogenic or Likely Pathogenic Variant

^1^ Adjusted for personal history of non-breast cancer and family history of breast cancer

### Screening adherence in year 2 and year 3

We also evaluated continuous adherence to BC screening for up to three years (Table 2). Breast MRI rates in the P/LP group were only slightly higher than the Empiric group in years two and three, with 38% (8/21) in the P/LP group and 35% (25/72) in the Empiric group undergoing MRI in year two (*p* = 0.980) and 36% (4/11) in the P/LP group and 20% (7/28) in the Empiric group undergoing MRI in year three (*p* = 0.418). Mammogram rates were higher for both groups and higher in the P/LP group than the Empiric group, with 78% (18/23) in the P/LP group and 51% (37/72) in the Empiric group undergoing mammograms in year 2 (*p* = 0.042) and 73% (8/11) and 43% (17/40) undergoing mammograms in year 3 (*p* = 0.098). For all three years of follow up, the P/LP group had a 30% higher adherence to mammography screening than the Empiric group.

Additional analyses were performed to measure screening over time for the entire study cohort. Adherence to MRI screening declined significantly, with the odds of adherence decreasing by 66% for each additional year of follow-up (OR = 0.34, *p* < 0.001). Similarly, adherence to mammography screening decreased significantly with increasing follow-up duration, corresponding to a 68% reduction in the odds of adherence per year (OR = 0.32, *p* < 0.001).

### Other factors associated with breast screening

We analyzed whether BC screening adherence was associated with demographic factors such as race/ethnicity, age, lifetime BC risk estimate, cancer history, FDR family history of BC, insurance status or hospital site.

Women who adhered to MRI screening recommendations within one year were significantly older than those who did not adhere to recommendations (median age of 47.4 versus 43.7, respectively) (*p* = 0.01); age was not found to be associated with likelihood of MRI screening beyond year one. Lifetime BC risk estimate was also associated with MRI adherence. Women adhering to MRI recommendations for two consecutive years had a higher median lifetime BC risk estimate than those who did not adhere (28.6% vs. 23.3%, *p* = 0.004). Race/ethnicity, cancer history, family history, insurance status, and hospital site were not identified to be associated with MRI adherence.

We also found that adherence to mammogram in one year was associated with insurance type—92% (11/12) of individuals with HMO obtained a mammogram within the first year compared to 67% (70/104) of patients with PPO and 54% (24/45) of women within the LA General safety-net insurance system (*p* = 0.045). Insurance type was not associated with the likelihood of screening mammogram beyond year one. Women who underwent a mammogram in year 1 had a higher median lifetime BC risk than those who did not have a mammogram (25.7% vs. 23.2%, *p* = 0.029), and this finding extended to year 2 (25.7% vs. 23.1%, *p* = 0.029). There were no additional demographic factors associated with differences in mammogram adherence.

### Breast biopsy, breast cancer diagnoses, and risk reducing mastectomies

The mean follow-up period for the study population was 2.24 years. Over this period, 8/44 (18%) of P/LP group and 29/117 (25%) of the Empiric group underwent at least one breast biopsy. Six women were diagnosed with either invasive BC or DCIS, including five in the Empiric group (5/117, 4.3%) and one in the P/LP group (1/44, 2.3%). All six women were adherent to mammogram, breast MRI, or both within the first year and received their diagnoses within the first two years of follow-up. Of the invasive BCs, four were stage T1N0, requiring no chemotherapy. However, one BC was staged as mpT1b(sn)N1a, requiring chemotherapy, despite her adhering to both MRI and mammography. She currently has no evidence of disease. In the remaining 31 women undergoing biopsies, the highest risk lesion identified in each woman included three with lobular carcinoma in-situ, six with atypical hyperplasia, seven with hyperplasia without atypia, and the remaining 15 had only a fibroadenoma or other benign lesion. The only three women in the study who underwent RRM were in the Empiric group including one woman who developed BC and two women with lifetime BC risks of 23–24% who both had a history of breast biopsies, one with low-risk benign lesions and the other with atypical lobular hyperplasia.

## Discussion

Women with a moderately increased BC risk represent a largely understudied, growing population as more women pursue MGPT and receive BC risk assessment with empiric risk modeling. We sought to observe factors associated with obtaining MRIs and mammograms following GC in an ethnically diverse cohort of moderate risk patients seen at a university medical center and a safety-net hospital. We found that in the first year GC, the presence of a P/LP variant was the strongest predictor of screening behavior.

In our cohort, 58% in the P/LP group obtained breast MRI within one year compared to 37% in the Empiric group. Rate of mammograms was also much higher in the P/LP group (88%) compared to the Empiric group (58%). Women in the P/LP group also obtained their BC screenings sooner than those in the Empiric group. A logistic regression showed that women in the P/LP group were 2.29 times as likely to obtain MRI within one year than the Empiric group, and 5.27 as likely to obtain mammogram. When adjusting for having a FDR with BC or history of non-BC, these differences were even more pronounced, with the P/LP group 3.18 times as likely to obtain breast MRI and 7.04 times as likely to obtain a mammogram, again showing the strong impact of the positive genetic test. The effect of having a FDR with BC or prior cancer history was not significantly associated with screening adherence.

Previous research has reported on MRI adherence for women across different BC risk stratifications and has also found a strong impact of a positive genetic test. A multicenter cohort study conducted by Naghi et al. administered surveys among women who had GC and MGPT. Compared to a MGPT negative group with lifetime BC risks < 20%, moderate-risk P/LP variant carriers were more than four times as likely (OR = 4.12 [95%CI, 1.10-14.35]; *P* = 0.03) to have undergone breast MRI during the four year study period and women with an empiric risk of BC ≥ 20% were more than twice as likely to obtain breast MRI (OR, 2.46 [95%CI, 1.40–4.32]; *P* = 0.002) [11]. However, in the first year of follow up, just 2 of 16 (12.5%) with a moderate-risk P/LP variant and just 20 of 146 (13.7%) of women with ≥ 20% lifetime BC risk underwent MRI, compared to 19 of 43 (44.2%) of *BRCA* carriers.

Other studies have found higher levels of breast MRI adherence but have longer follow-up periods and were conducted with surveys. Cragun et al. surveyed female *ATM* and *CHEK2* P/LP carriers and found 9/10 (90%) had at least one breast MRI after testing positive, though the follow-up period was over several years [12]. Similarly, Esani et al. reported that 108/123 (88%) of women at ≥ 20% lifetime BC risk had one MRI in the medical record over a follow-up period of 4.25 years [18]. Vysotskaia et al. also surveyed women with P/LP variants in *PALB2*,* ATM*,* CHEK2*,* NBN*,* BRIP1*,* RAD51C*, and/or *RAD51D* about adherence to breast MRI. Over a follow-up period of 5 months to 2.5 years, 47/66 (71%) of P/LP variant carriers reported having had at least one MRI and the remaining 17/66 (26%) reported that they planned to get an MRI in the future [14]. These studies provide useful exploration of the topic but may have been subject to recall and response bias.

There are factors that may be associated with breast cancer screening adherence that our study was not able to assess such as comprehension of recommendations, issues with planning and limited time available for screening, cost or insurance coverage for screening, anxiety, discomfort, desire to avoid contrast dye for breast MRI, lack of knowledge on how to schedule an appointment, excessive delays in getting a screening appointment due to wait time, or contraindication based on medical devices or other medical history. Furthermore, social determinants of health such as lack of transportation may impact adherence. Future studies such as questionnaires and interviews in among women with increased risk could help identify reasons for non-adherence to screening, which could guide a targeted intervention to improve screening.

In addition to using the EMR to collect data, studying screening behavior over multiple time periods enabled us to observe that screening rates decline over time. Only 36% of women in the P/LP group and 20% of women in the Empiric group obtained breast MRIs for three consecutive years. Naghi et al. also analyzed continuous MRI adherence over three years with similar results—6/16 (37.5%) moderate-risk P/LP variant carriers and 33/146 (22.6%) of women with ≥ 20% lifetime BC risk obtained MRI [11]. Our analyses showed that both breast MRI and mammogram adherence decreased significantly with increasing follow-up duration, which may be attributable to waning salience of the initial recommendation. Furthermore, negative experiences during an initial mammogram or breast MRI such as discomfort and unanticipated out-of-pocket costs may discourage participation in subsequent screening. Finally, those with a positive genetic test may feel an urgency to obtain breast screening, reflected in the observation that they had a quicker uptake of screening, but their level of concern may decrease after normal screens and after they adjust to their genetic result, potentially leading to reduced commitment to screening in subsequent years. In our study, we defined adherence in years two and three as predicated on obtaining screenings in each previous year; therefore, women who decide to obtain breast MRIs at intervals larger than one year are reported in our results as non-adherent. It is more difficult to be considered a continuous screener through three years than obtaining what is often a woman’s first breast MRI shortly following GC.

Our finding that breast MRI screening rates were lower than mammogram screening rates aligns with previous research. Knerr et al. also reviewed medical records to determine screening adherence for 140 women with P/LP variants in BC susceptibility genes, including 18 *CHEK2* and 5 *ATM* carriers. Their study showed that, on an annual basis, women were just 48% adherent to mammogram and 34% adherent to breast MRI, with a combined average adherence to both modalities of only 19% [13]. Mammogram adherence is likely higher than MRI because mammograms do not require contrast dye injection or induce claustrophobia, take less time, require less coordination with referrals and pre-authorizations, and have proven cost-effectiveness [19, 20]. Furthermore, patients may be more likely to understand the recommendation for mammograms better than the recommendation for breast MRIs [21].

Besides the presence of a P/LP variant, most other characteristics had little or no effect on adherence to screening. However, having a higher lifetime BC risk estimate was associated with undergoing mammography in the first and second year and was associated with two years of adherence breast MRI. While statistically significant, these lifetime risk differences between screeners and non-screeners were small. Another study evaluating the effect of lifetime risk estimate was conducted by Do et al. in a military population of 1,052 empiric risk women qualifying for MRI. In this study, just 251 (24%) obtained at least one breast MRI over a three year follow-up period; women with Tyrer-Cuzick risk estimates of 20–24%, 25–29%, 30–39%, and ≥ 40% were 16%, 24%, 37%, and 51% compliant with breast MRIs, respectively [21]. Consistent adherence to screening may indeed be more likely among women at higher empiric risk, but further research is needed to determine how the risk calculation impacts screening behavior. Insurance status was a predictor of adherence to mammography in year one, with HMO patients having the highest adherence (92%) compared with PPO patients (67%) and LA General (54%). While the number of HMO patients was low (*N* = 12), adherence could be higher in this group for health care system reasons, because an approved authorization usually triggers appointment scheduling. Additionally, HMO patients value receiving care at a university medical center, which is not always possible because their insurance often redirects mammography to community practices, so they may be more likely to follow through with their appointments. Interestingly, in our diverse population, patient demographic characteristics such as race/ethnicity and hospital site were not significantly associated with adherence to screening.

While medical record review is a strength of the study because it eliminates biases introduced by survey administration, it is also a limitation because patients with ongoing care at our facility but the absence of a screening report on file does not exclude the possibility they may have had screening at an outside institution. While the size of our P/LP group is larger than other cited studies of screening adherence, it is still relatively small, given the rare nature of identifying women with P/LP variants. The number of women eligible for analysis through three years was reduced, both because some patients had GC recommendations made in the later years of the study period (i.e. 2023 and 2024) and others did not have any ongoing care documented in the medical record and were thus censored in our analysis.

Though our study aimed to understand factors associated with BC screening adherence, it is imperative that we learn more about the reasons for screening behavior before implementing an intervention to improve screening. Conley et al. found that facilitators of BC screening for women with greater than 20–25% lifetime BC risk included patient knowledge and understanding of screening options, self-advocacy, social support, belief in the importance of screening, and trust in healthcare providers, while barriers to screening included financial burden, lacking or conflicting provider recommendations, and logistical challenges [20]. Future studies will expand on this research, allowing for interventions to improve access and adherence.

## Conclusion

Our study included two groups of women, both with a moderately increased breast cancer risk; however, women who tested negative on multigene panel testing (MGPT) demonstrated lower adherence to screening recommendations than women with a moderate-risk P/LP variant. By assessing adherence over subsequent years, we found that mammogram rates are much higher than breast MRI rates and that screening declines over time. Genetic testing and cancer risk assessment are extremely important for guiding screening recommendations. Ultimately, the outcome of early detection is directly linked to adherence. Future research should aim to improve screening adherence, leading to better outcomes in this important, growing population.

## Data Availability

The datasets generated during the current study are not publicly available since they include patient identifiers but could potentially be de-identified and available from the corresponding author on reasonable request.
